# Potential effect modification of RTS,S/AS01 malaria vaccine efficacy by household socio-economic status

**DOI:** 10.1186/s12889-021-10294-x

**Published:** 2021-01-28

**Authors:** Stephaney Gyaase, Kwaku Poku Asante, Elisha Adeniji, Owusu Boahen, Matthew Cairns, Seth Owusu-Agyei

**Affiliations:** 1 Kintampo Health Research Centre, Ghana Health Service, Kintampo, Ghana; 2 Tropical Epidemiology Group, London School of Hygiene and Tropical Medicine, London, UK; 3 Institute of Health Research, University of Health and Allied Sciences, Ho, Volta Ghana

**Keywords:** Socio-economic status, Malaria, Vaccine, Protective efficacy, Malaria incidence

## Abstract

**Background:**

In the phase III RTS,S /AS01 trial, significant heterogeneity in efficacy of the vaccine across study sites was seen. Question on whether variations in socio - economic status (SES) of participant contributed to the heterogeinity of the vaccine efficacy (VE) remains unknown.

**Methods:**

Data from the Phase III RTS,S /AS01 trial in children aged 5–17 months in Kintampo were re-analysed. SES of each child was derived from the Kintampo Health and Demographic Surveillance System, using principal component analysis of household assets. Extended Cox regression was used to estimate the interaction between RTS,S/AS01 VE and household SES.

**Results:**

Protective efficacy of the RTS,S/AS0 vaccine significantly varied by participant’s household SES, thus increase in household SES was associated with an increase in protective efficacy (*P*-value = 0.0041). Effect modification persisted after adjusting for age at first vaccination, gender, distance from community to the health facility, child’s haemoglobin level, household size, place of residence and mothers’ educational level.

**Conclusion:**

Household SES may be a proxy for malaria transmission intensity. The study showed a significant modification of the RTS,S/AS01 malaria vaccine efficacy by the different levels of child’s household socio - economic status.

**Trial registration:**

Efficacy of GSK Biologicals’ candidate malaria vaccine (25049) against malaria disease in infants and children in Africa. NCT00866619 prospectively registered on 20 March 2009.

## Background

Significant attempts have been made to address the burden of malaria in endemic countries. Despite signs of substantial global progress, the burden in some developing countries remains high, resulting in a negative impact on health and livelihoods. Ghana remains one of the countries in Africa with the highest burden of malaria, with a reported increase in malaria cases in 2018 compared to 2017. Malaria accounted for close to 34% of total outpatients’ cases, 55.6% of all admissions and 54.6% of deaths in children who were under 5 years of age. *Plasmodium falciparum* remains the most prevalent malaria parasite in Ghana and reflects the sub–Saharan Africa endemicity, where it accounted for over 99% of malaria cases in 2018 among young children [1–3].

The RTS,S /AS01 malaria vaccine (Mosquirix) has been developed as an additional tool for malaria control. In the pivotal phase III RTS,S /AS01 malaria vaccine trial, the incidence rate for all episodes of clinical malaria was 0.69/person-years in the RTS,S /AS01 group and 1.17/person-years in the control group in the per-protocol population based analysis on the primary case definition after 18 months follow-up. This resulted in a protective efficacy of 46% (95% CI: 42, 50%) against clinical malaria in children aged 5 to 17 months. Within the same age group, the incidence rate of clinical malaria based on the per-protocol definition for the Kintampo Health Research site was 1.01/person-years and 1.85/person-years in the RTS,S/AS01 and control groups respectively. This resulted in the protective efficacy of 47% (95% CI: 39.1, 54.2%) for the research site.

However, significant heterogeneity in vaccine efficacy ranging from 40 to 77% (interaction test, *p*, 0.001) was observed across study sites [4]. The reported heterogeneity in VE was investigated using malaria transmission intensity defined as the occurrence of clinical malaria within the control group at a given site. The heterogeneity of the vaccine efficacy across study sites and it being shown to be higher at low transmission areas and lower at higher transmission areas [4] suggest that, occurance of clinical malaria alone could not have accounted for this heterogeneity but other factors such as receipent’s socio-economic status could have influenced the results of the trial. Other research findings have showed the modification of one’s socio - economic status and efficacy of vaccines. A study by Gosselin et al. showed that SES seemed to have influence on the efficacy of rotavirus vaccine. Children living with high rates of low-income families was shown to have had a significant lower VE compared to children living with lower rates of low-income families [5]. Again, Lopman et al. also showed that rotavirus vaccine prevented gastroenteristics diseases by 93, 86 and 51% in high, middle and low SES repectively [6]. Also, findings of a population-based case-control cohort analysis by Hammer et al. during the pertussis outbreak in 2004/2005 also found the effectiveness of the pertussis vaccine being influenced by SES [7]. Though the RTS,S/AS01 Phase III clinical trial was conducted in a standardized clinical and laboratory methods across study sites, the critical question of whether variations in socio - economic status of participant contributed to the heterogeinity of the RTS,S/AS01 VE remains unknown [4].

Kintampo Health reasrch centre which was part of the Phase III clinical trial is a peri-urban with malaria transmission being high, with seasonal peaks and troughs [8]. Again, individual household socio-economic indicators in this study site have also been showed to be an indicator for malaria exposure, thus malaria exposure being low for high SES and high for low SES [9]. This makes the study site ideal to examine the modification of SES on the RTS,S/AS01 VE.

The objective of the study was to invitigate the variability of the RTS,S/AS01 malaria vaccine, using SES as a proxy for malaria exposure in the Kintampo site in Ghana.

A description of the demographic characteristics and the incidence of clinical malaria episodes are described in the results section. We discussed and conclude that, Socioeconomic status was an effect modify of the RTS,S/AS01 malaria vaccine efficacy.

## Methods

### Data

The methods and results of the original phase III RTS,S /AS01 clinical trial and this study have been reported according to CONSORT guidelines [10] . Data for this research was obtained from the Phase III RTS,S /AS01 malaria vaccine trial (NCT00866619) involving children aged 5–17 months. The children were enrolled in the Kintampo Health Research Centre study area located in the Kintampo North Municipality and Kintampo South District in the Bono-East Region of Ghana. This study area has been endowed with a Health and Demographic Surveillance System (HDSS) that collects socio-demographic data on the population. The total surface area of these two districts is 7162 km^2^ and comprises of about 156,145 residents (Kintampo Health Research Annual Report, 2016).

The methods deployed in the main trial have been published elsewhere [11]. In summary, the trial was a randomised, double-blind controlled trial of the candidate RTS,S/AS01 malaria vaccine, conducted at 11 sites in seven sub-Sahara African countries with varied malaria transmission. The trial was designed to evaluate malaria vaccine efficacy, safety and immunogenicity in infants and young children. A randomisation ratio of 1:1:1 was used with the first group receiving three doses of the RTS,S/AS01 at monthly intervals, followed by a booster dose at 18 months after completing the primary vaccinations; the second group received the RTS,S /AS01 primary vaccination series without the booster and the third group received comparator vaccines.

For this analysis, data from children in the first and second groups, that is all children who received at least one dose of RTS,S/AS01 malaria vaccine, were combined and compared with the third group (control group) in the ratio of 2:1. A clinical malaria episode was defined based on a participant presenting at the health facility with axillary temperature ≥ 37.5 °C and presence of *Plasmodium falciparum* asexual parasitaemia greater than zero parasites/μL. To avoid counting the same episode more than once, any episode of clinical malaria documented within 14 days of treatment for a previous episode was discounted. A total of 1002 children who were followed up to 18 months after completing the primary vaccination series was included in the analysis.

Data on household SES of each participating child was obtained from the Kintampo Health and Demographic Surveillance System (KHDSS). The KDDSS process involved administering a standardised SES questionnaire to each household head or in their absence, an adult resident member of the household who was present on the day of the interview. All respondents were questioned about ownership of household assets including television, radio, bicycle, motorcycle, refrigerator, car, ownership of farmland, electricity at homes. Other SES variables that they enquired about included the type of building material, ceiling in the house, room floor cover, number of sleeping rooms, home ownership, source of drinking water, type of toilet facility and household ownership of domestic animals such as poultry, goat, sheep, pigs, cattle among others.

### Statistical methods

In this analysis, SES was assessed using household assets (ownership of television, radio, motorcycle, refrigerator, car, farmland, mattress, internet, satellite disc, electricity at home, type of material used for roofing, building material used for room floor and walls, source of water, toilet facility and ownership of domestic animals such as pigs, sheep among others). Both categorical and continuous data were collected during interviews on household assets. Questions based on these household assets were used in generating assets scores for each household. An overall wealth index was computed for each participant, using principal component analysis (PCA) as described by Vyas et al. [12]. The first component was used in generating household wealth index. Participants were grouped by wealth index that is based on their SES, with tertiles used to classify a child’s SES as low, medium or high.

Incidence of clinical malaria was estimated as the total number of clinical malaria episodes experienced by each child after the primary vaccination series and until 18 months post-vaccination follow-up. An extended Cox regression model [13] was used to estimate the hazard ratio comparing RTS,S/AS01 recipients with the control groups. Vaccine efficacy was estimated as one minus the hazard ratio, expressed as a percentage. The primary comparison of interest was the interaction between the study group and household socio-economic status, with the Wald test of the interaction parameter used to assess the evidence against the null hypothesis of no interaction.

A set of variables: haemoglobin status, classified into < 10 g/dL as anaemic and ≥ 10 g/dL as normal; distance between the resident community of a participant and the nearest health facility based on the road networks, classified into < 5 km, 5-10 km, 11-15 km and above 15 km; place of residence, classified as either rural or urban; household (HH) size, classified as < 5 persons per HH or above 5 persons per HH; age group at first vaccination, classified as 5–8 months, 9–12 months, 13–17 months; care giver’s educational level, classified as none, primary, secondary or higher and gender of the respondent. These were considered as potential confounders for inclusion in the final model.

Vaccine-preventable disease incidence (VPDI) for each SES level was estimated based on a weighted least-squares regression model with robust standard error [14].

The analysis was carried out using STATA 14 (StataCorp, TX, USA) and R version 3.5.0.

## Results

### Study participants

Of the 1002 children included in this analysis, 668 were assigned to the RTS,S/AS01 malaria vaccine group and 334 to the control group. Males constituted 51.4% of the total participant’s data analysed. Majority of the children were within the ages thirteen and seventeen months when they were enrolled at vaccination. Distribution of the different levels of SES was similar between the two groups; ten of the participants, however, did not have information on their socio-economic status (Table 1).

**Table 1:** Comparing participants basic demographics status between the groups

Attribute	RTS,S /AS01 Group (***N*** = 668) % (n)	Control Group (***N*** = 334) % (n)
**Age at Vaccination**		
5–8 months	26.7 (178)	23.7 (79)
9–12 months	20.8 (139)	25.7 (86)
13–17 months	52.5 (351)	50.6 (169)
**Gender**		
Male	53.6 (358)	47.0 (157)
Female	46.4 (310)	53.0 (177)
**Socio - Economic Status**		
Low	33.8 (226)	33.8 (113)
Medium	34.4 (230)	32.0 (107)
High	31.0 (207)	32.6 (109)
Missing	0.7 (5)	1.5 (5)

#### Descriptive statistics of other demographic factors by household socio - economic status

More than half of the children classified within the medium and low SES had caregivers with no basic school education (that is no formal education), compared to the majority of children classified within the high SES who had caregivers with secondary or higher education. The number of persons per household was evenly distributed between less than five and above five persons per household. Majority of households in the rural communities were those with low SES, compared to those with high SES who were more frequently from urban communities. More than half of the children from the low and medium SES were anaemic, compared to the other half of children within the high SES with normal haemoglobin levels. About 5% of the participants care givers information on educational status were missing. Again, there were no information on place of residence for about 7% of the participants in the study (Table 2).

**Table 2:** Summary statistics of participant basic demographics characteristics by household socio-economic status

	Household Socio-economic status		
Attribute	Low (***N*** = 339) % (n)	Medium (***N*** = 337) % (n)	High (***N*** = 316) % (n)
**Care Giver’s educational status**			
None	64.3 (218)	50.4 (170)	34.5 (109)
Primary	12.1 (41)	19.0 (64)	18.7 (59)
Secondary / Higher	13.6 (46)	20.8 (70)	43.0 (136)
Missing	10.0 (34)	9.8 (33)	3.8 (12)
**Household Size**			
Above 5 person/HH	48.7 (165)	50.7 (171)	42.4 (134)
Less than 5 person/HH	51.3 (174)	49.3 (166)	57.6 (182)
**Place of Residence**			
Rural	80.5 (273)	64.7 (218)	39.9 (126)
Urban	14.4 (49)	28.2 (95)	50.0 (158)
Missing	5.0 (17)	7.1 (24)	10.1 (32)
**Haemoglobin Status (Child)**			
Normal	34.2 (116)	41.2 (139)	51.3 (162)
Anaemic	65.8 (223)	58.8 (198)	48.7 (154)

**Fig. 1:**
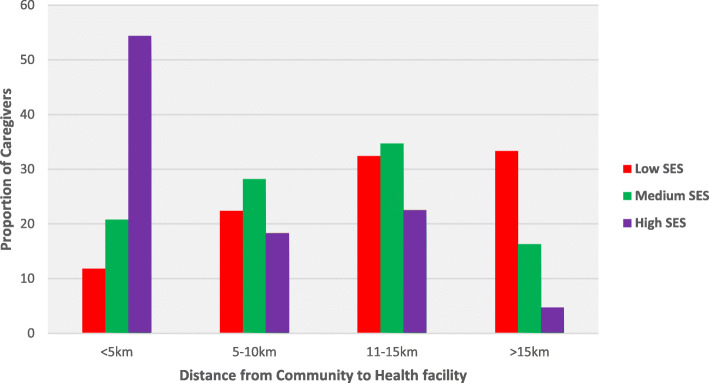
Description of distance/km travelled by paticipants from the community to the nearest health facility in the study area

Majority of participants in households with high socio-economic status lived closer to a health facility compared to the majority of the participants from households with low SES who lived more than 10 km away from a health facility (Fig. 1).

### Malaria incidence by household SES group

A total of 2651 distinct episodes of clinical malaria were recorded over the 18 months’ period of follow-up. Overall, 1464 episodes were recorded in 828.41 person-years for the 668 children in the RTS,S/AS01 group and 1187 episodes in 399.76 person-years for the 334 children in the control group. The incidence rate ratio (IRR) of clinical malaria in the study was 0.58 (95% CI: 0.52, 0.66) and the protective efficacy was estimated as 42% (95% CI: 0.34, 0.48)].

For both treatment group, there were 1153 distinct episodes of clinical malaria in 410.13 person-years, 914 distinct episodes of clinical malaria in 415.00 person-years and 558 distinct episodes of clinical malaria in 391.65 person-years experienced by children from the household with low, medium and high socio-economic status respectively. In the intervention group, the incidence rates of clinical malaria for the low, medium and high SES were 2.40 (95% CI: 2.22, 2.59), 1.88 (95% CI: 1.73, 2.05) and 0.97 (95% CI: 0.86, 1.09) per child years at risk respectively. In the control group, the corresponding incidence rates of clinical malaria for each level of SES were 3.67 (95% CI: 3.3, 4.00), 2.89 (95% CI: 2.62, 3.20) and 2.37 (95% CI: 2.12, 2.65) (Table 3).

**Table 3:** Comparing the incidence of clinical malaria among the treatment groups

	RTS,S /AS01 Group				Control Group			
Household Socio-economic status	Total Episode	Person time at Risks (years)	Rate	95% CI	Total Episode	Person time at Risks (years)	Rate	95% CI
**Low**	663	276.48	2.40	2.22–2.59	490	133.64	3.67	3.35–4.00
**Medium**	533	283.43	1.88	1.73–2.05	381	131.57	2.89	2.62–3.20
**High**	256	264.26	0.97	0.86–1.09	302	127.39	2.37	2.12–2.65
**Missing**	12	4.23	2.8	1.61–4.99	14	7.15	1.96	1.16–3.30

Rate is Episodes of clinical malaria per person-years at risk

**Fig. 2:**
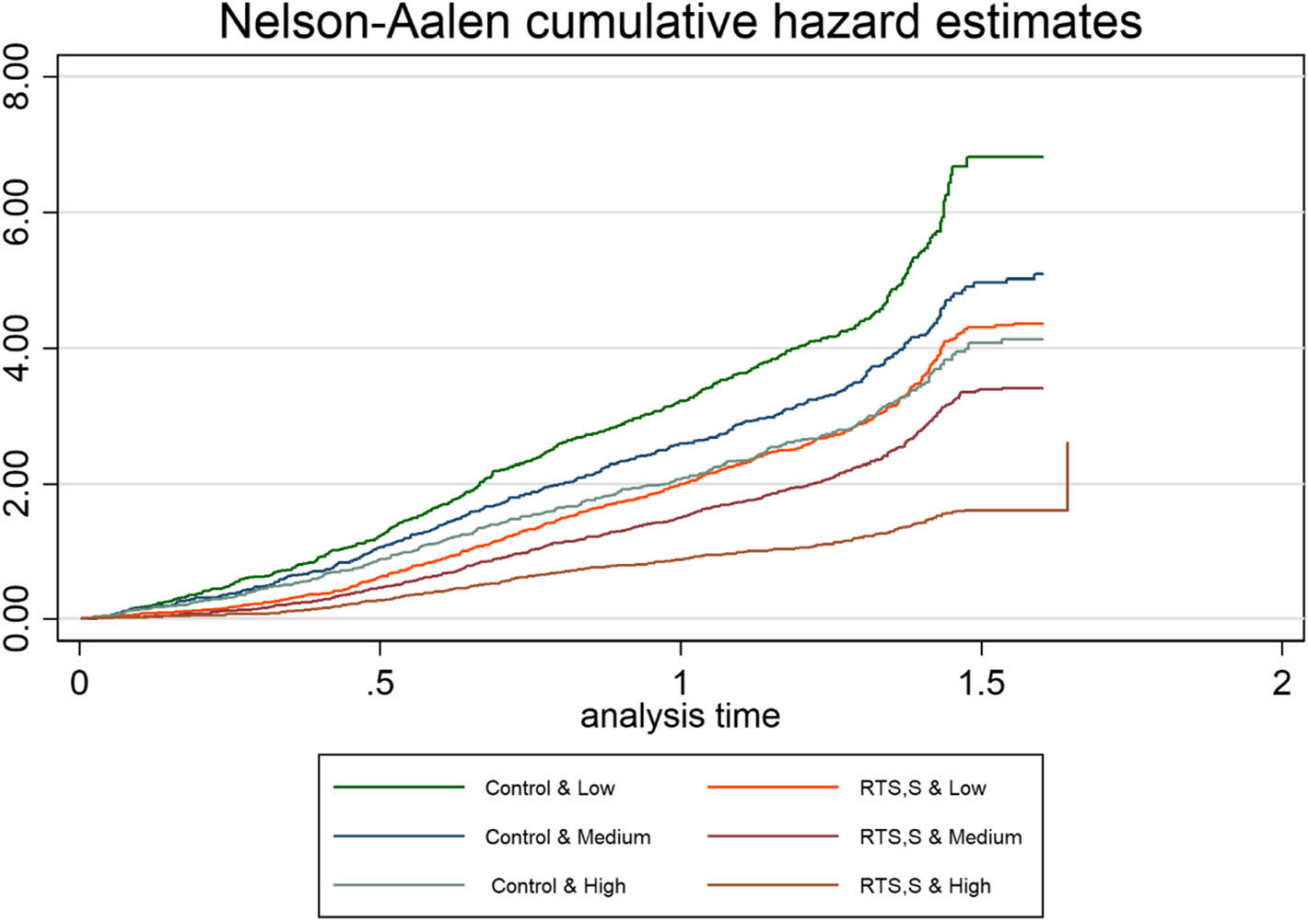
Cumulative hazard of the effect of child’s HH SES on the RTS,S /AS01 malaria vaccine efficacy over the follow-up period

The rate of clinical malaria kept increasing over the follow-up period, though the increase was higher for children in the control group compared to the RTS,S/AS01 group. However, the rate of clinical malaria for children from high SES in the control group and children from low SES in the RTS,S/AS01 group seemed similar over time (Fig. 2).

Though the protective efficacy of the RTS,S /AS01 malaria vaccine for children in the low (VE = 36, 95% CI: 25, 45%) and medium SES class (VE = 36, 95% CI: 22, 47%) were similar, the protective efficacy was about twice as high among children in the high SES group (VE = 61, 95% CI: 49, 70%). There was strong evidence for effect modification, (*p* = 0.0041).

The vaccine-preventable disease incidence over 18 months of follow-up in the three different SES groups was 1268.5, 1015.3 and 1401.8 per 1000 children vaccinated for children from the low, medium and high SES respectively. The low vaccine efficacy in the group with the highest incidence (low SES) and the highest vaccine efficacy in the group with the lowest malaria incidence (high SES) resulted in similar VPDI in these two groups.

After adjusting for distances from communities to the health facility, child’s haemoglobin level, gender of the child, household size, place of residence and mother’s educational level, age at first vaccination, the efficacy of the vaccine still varied with SES, (*p* = 0.0029). The protective efficacy still decreased as the child’s SES decreased. Again, the efficacy of the vaccine for children from the high SES was about twice the efficacy of the vaccine for children from the low SES (Table 4).

**Table 4:** Incidence rate ratio of clinical malaria for each SES stratum

Parameter	Unadjusted			Adjusted		
	Protective Efficacy (Percent)	95% CI	***P***-Value	Protective Efficacy (Percent)	95% CI	***P***-Value
Low SES	35.65	25.04–44.76	< 0.001	32.64	21.75–42.01	< 0.001
Medium SES	35.75	22.28–46.89	< 0.001	36.11	22.89–47.06	< 0.001
High SES	60.83	48.79–70.03	< 0.001	60.01	47.94–69.29	< 0.001

Wald test and *P*-value for an interaction effect between participant’s SES and the treatment groups for the unadjusted model was (χ^2^ = 10.99, *P*-value = 0.0041) and was (χ^2^ = 11.74, *P*-value = 0.0028) for the adjusted model

Twenty-two percent (217) of children in the entire cohort did not experience any clinical malaria episodes throughout the 18-month follow-up period. For children without clinical malaria episodes throughout the 18-month follow-up period, 80.6% (175) were in the intervention group whiles 19.3% (42) were in the control group.

## Discussion

In this study, we explored the potential interactions between RTS,S/AS01 vaccine efficacy and household socio-economic status. In the pivotal Phase III clinical trial, VE was seen to be homogeneous across site. Albeit, this homogeneity was not explained by transmission, the study found the efficacy of the vaccine to be higher in areas with low clinical malaria incidence. Similarly, studies have shown that clinical malaria incidence is low for households with high SES compared to household with low SES within the Kintampo health research study area [4, 9]. SES was therefore treated as a proxy for malaria transmission intensity to explore the potential interaction with malaria RTS,S/AS01 vaccine.

VE against clinical malaria was seen to have a significant heterogeneity among the different study participant groups. This heterogeneity was, however, seen to be driven by the participant’s household socio-economic status; individuals of higher socio-economic status experienced markedly lower malaria incidence rates. Thus, VE was higher among SES group with lower exposure resulting in better protection.

This study finding, demonstrating that RTS,S /AS01 malaria vaccine efficacy varies according to the socio-economic status of the recipient, is consistent with the broad patterns observed in the Phase III RTS,S/AS01 trial. In that study, VE was higher in low malaria transmission settings than in higher malaria transmission settings [4]. Again the findings in this study are in line with the Phase 2 trials, which also found the efficacy of the RTS,S/AS01 malaria vaccine decreasing with increasing exposure [3]. In the present study, individuals of high SES, who were at much lower overall risk of malaria, experienced markedly higher vaccine efficacy. The result is also consistent with other research findings, which found a positive association of other vaccines and one’s socio-economic status [5–7, 15]. One of these studies by Lopman et al. was on children aged between 6 and 23 months, demonstrated that rotavirus vaccine prevented 93, 86 and 51% of severe rotavirus gastroenteritis in high, middle and low SES respectively [6]. Another study carried out in Quebec using the rotavirus vaccine among children less than 3 years of age also found children from a neighbourhood with a higher rate of low-income families having a significantly low VE compared to children from a neighbourhood with a lower rate of low-income families (30% vs. 78%, *p* = 0.027) [5]. A study that investigated the effectiveness of new group B strain-specific meningococcal vaccine in New Zealand also found significantly higher meningococcal rates for higher levels of deprivation (*p* < 0.001) [15]. Furthermore, a study that investigated Pertussis vaccine found the vaccine to be 79, 86, 83 and 94% for children in the first, second, third and fourth quartiles of houses (an indicator of SES) respectively (*p* = 0.03) [7].

Reasons that might contribute to the low efficacy among low SES include housing quality, level of exposure, reduction in vaccine immunogenicity, infectious agent, one’s susceptibility to agent among others. Poor households are likely to live in rural areas with poor housing quality, poor sanitation and high malaria breeding sites that will lead to a higher risk of transmission [9, 16–18]. In this study, more than half of the population with low or medium socio-economic status reside in the rural parts of the study area, in houses built with mud and straw roofing that create easy access routes for infective malaria mosquitoes. Their homes are usually located in areas with unkept bushes, no proper drainage and without designated places for waste disposal. All these facilitate congenial environments for breeding malaria mosquitoes [9, 16–18].

Distances from rural communities to health facilities have been associated with the risk of malaria infection in places with high malaria transmission [19]. Those living far from health facilities find it more difficult in accessing the health-care facility. Majority of participants in this study who were within the low SES households lived far from health facilities with poor access to the main facility.

Though SES could be a malaria-risk factor that modifies VE, other biological factors including immune responses could also explain the lower VE among participants of low SES. Low haemoglobin has been reported to be associated with poor immune responses [20, 21]; Our study findings document a higher proportion of participants, i.e. more than half of the children from the low SES had low haemoglobin.

To ascertain whether the interactive effect between RTS,S/AS01 malaria vaccine efficacy and caregiver’s household SES was real rather than spurious, the model adjusted for the gender of the child, caregiver’s educational status, distance from the household to a health facility, the size of the household, haemoglobin status of the child, place of residence, age at first vaccination. The interaction effect remained statistically significant.

There are, however, some caveats to this finding. First, this study uses SES as a proxy to a true measure of exposure given that direct measurements, such as entomological inoculations are expensive and imprecise due to the microheterogeneity of malaria transmission [22]. Again, 0.9% of the participants did not have SES information and were therefore not eligible for inclusion in the analysis. Excluding them from the analysis did not bias the results as their exclusion did not alter the distribution of SES categories between the RTS,S/AS01 and control group. Other residual confounding could have resulted from variables used in the SES estimation but these were not measured.

## Conclusion

This study showed a significant modification of the RTS,S/AS01 malaria vaccine efficacy by the different levels of child’s household socio-economic status. The effect of the modification remained statistically significant after adjusting for other potential confounders.

This study suggests that the impact of the RTS,S/AS01 malaria vaccine may be minimal in groups that most need it and that other measures to ensure clinical malaria incidence remains low will be of great public health importance.

## Data Availability

The data set used in this analyses were obtained from the Clinical Trials Team and GSK data archival team as well as the Management Team of Kintampo Health and Demographic Surveillance System. They are still custodians who can be approached for access to data used.

## Abbreviations

SES: Socio - economic status
HH: Household
GSK: GlaxoSmithKline
HDSS: Health and Demographic Surveillance System
ITT: Intention to Treat
KHDSS: Kintampo Health and Demographic Surveillance System
PCA: Principal Component Analysis
VE: Vaccine efficacy
VPDI: Vaccine preventable disease incidence
